# Chrysoeriol Improves the Early Development Potential of Porcine Oocytes by Maintaining Lipid Homeostasis and Improving Mitochondrial Function

**DOI:** 10.3390/antiox13010122

**Published:** 2024-01-19

**Authors:** Chao-Rui Wang, Xiu-Wen Yuan, He-Wei Ji, Yong-Nan Xu, Ying-Hua Li, Nam-Hyung Kim

**Affiliations:** Guangdong Provincial Key Laboratory of Large Animal Models for Biomedicine, School of Biotechnology and Health Sciences, Wuyi University, Jiangmen 529000, China; lindbergh0329@163.com (C.-R.W.); jihewei0608@163.com (H.-W.J.);

**Keywords:** chrysoeriol, porcine, oocyte maturation, lipid homeostasis, mitochondrial function

## Abstract

Our previous study established that chrysoeriol (CHE) can reduce reactive oxygen species (ROS) accumulation, apoptosis, and autophagy in vitro culture (IVC) of porcine embryos. However, the role of CHE in oocyte maturation and lipid homeostasis is unclear. Herein, we aimed to elucidate the effect of CHE on porcine oocyte competence in vitro maturation (IVM) and subsequent embryo development. The study chooses parthenogenetic activated porcine oocytes as the research model. The study revealed that the cumulus expansion index and related gene expressions are significantly elevated after supplementing 1 μM CHE. Although there were no significant differences in nuclear maturation and cleavage rates, the blastocyst formation rate and total cell numbers were significantly increased in the 1 μM CHE group. In addition, CHE improved the expression of genes related to oocyte and embryo development. ROS was significantly downregulated in all CHE treatment groups, and intracellular GSH (glutathione) was significantly upregulated in 0.01, 0.1, and 1 μM CHE groups. The immunofluorescence results indicated that mitochondrial membrane potential (MMP) and lipid droplet (LD), fatty acid (FA), ATP, and functional mitochondria contents significantly increased with 1 μM CHE compared to the control. Furthermore, CHE increased the expression of genes related to lipid metabolism, mitochondrial biogenesis, and β-oxidation.

## 1. Introduction

After in vitro maturation (IVM), oocytes must undergo the in vitro culture (IVC) phase. Chrysoeriol (CHE), a flavonoid compound, has been proven to reduce oxidative stress, reactive oxygen species production, and porcine oocyte apoptosis and autophagy in IVC [1]. In addition, a previous study demonstrated that CHE can inhibit fat deposition and promote lipolysis in adipocytes by increasing free glycerol and fatty acid levels [2]. Moreover, CHE exhibits lipase inhibitory activity, which is why it can be used to treat diabetes [3]. Although how CHE enhances porcine oocyte development during the IVC stage has been studied, its effects on the early development of porcine oocytes in the IVM stage remain unclear.

Oocyte maturation is a highly complex process, essential in determining the developmental potential of embryos. Lipid metabolism, including lipogenesis and lipolysis, provides energy sources during oocyte maturation and embryo development [4]. Oocyte intracellular lipids are primarily stored in lipid droplets (LDs) as triglycerides (TG), and endogenous triglycerides are integral for energy metabolism during porcine oocyte in vitro maturation [5,6]. LDs originate from the endoplasmic reticulum, are storage reservoirs for lipids and proteins, and directly communicate with other organelles [7].

Oocytes maintain intracellular lipid homeostasis by balancing lipogenesis and lipolysis. During lipolysis, lipase in cumulus cells and oocytes metabolizes triglycerides, and the generated fatty acids (FAs) are further metabolized through β-oxidation to produce ATP in mitochondria [8]. Compared with the in vivo development of oocytes, oocytes that undergo in vitro maturation (IVM) are more prone to metabolic abnormalities and accumulating excessive lipid content. A previous study demonstrated that this effect is related to fatty acid binding protein (FABP) [9].

Porcine oocytes contain a high content of fatty acids and endogenous lipids, containing 161 ± 18 μg of endogenous lipids, significantly higher than other species (cattle: 63 ± 6 μg; sheep: 89 ± 7 μg) [10]. Thus, porcine oocytes are an excellent model for studying lipid and FA metabolism. Another reason behind the excessive lipid accumulation in porcine oocytes is that follicular fluid and cumulus–oocyte complexes (COCs) provide a fat-rich environment, which is harmful to oocyte development in vitro. Similar harmful events have also been reported during oocyte maturation in vivo, a prominent factor of infertility in women with obesity [11,12,13].

Strong evidence shows that mitochondria quality and quantity are vital for successful oocyte maturation, fertilization, and subsequent development of embryos. Except for the nucleus, mitochondria are animal organelles that contain genetic information called mitochondrial DNA (mtDNA) and are related to ATP production [14]. Mitochondria and LDs exhibit a close interaction. Previous studies have proven a molecular connection between LDs and mitochondria [6]. Therefore, evaluating mitochondrial function during the IVM stage of oocytes is necessary.

Herein, we hypothesize that CHE can improve the early development of porcine oocytes by maintaining lipid metabolism and mitochondrial function. Therefore, we evaluated the quality of early oocyte development through COC expansion, nuclear maturation, and oxidative stress levels. Then, related lipid metabolism and mitochondrial function indexes were detected. Finally, the developmental ability of embryos was evaluated based on total cell number, cleavage, and blastocyst formation rate during the IVC stage.

## 2. Methods and Materials

### 2.1. Chemicals

Unless otherwise specified, all reagents used in this study were purchased from Sigma-Aldrich (St. Louis, MO, USA). The porcine ovaries were obtained from a slaughterhouse (Jiangmen, China) and transported to the laboratory within two hours. In the study, we conducted experiments with five dose treatment groups: control (non-treatment), 0.01, 0.1, 1, and 5 μM CHE.

### 2.2. Porcine Oocyte In Vitro Maturation

Porcine ovaries were obtained from slaughterhouses and kept at 35 °C during transportation. Next, the follicles were aspirated with a syringe, and cumulus–oocyte complexes (COCs) were collected with numerous cumulus cell layers. Finally, 500 μL of IVM medium (M199 cell medium with 10 IU/mL of follicle-stimulating and luteinizing hormone, 0.91 mM sodium pyruvate, 10 ng/mL of epidermal growth factor, 1 μg/mL insulin, and 10% follicular fluid of porcine) was added to each well of the four-well plate (Nunc, Roskilde, Denmark), and approximately 50 oocytes were cultured in each well. The selected COCs per group were then cultured at 38.5 °C in a 5% CO_2_ atmosphere for 42–44 h with control, 0.01, 0.1, or 1.5 μM CHE.

### 2.3. Evaluation of Cumulus Cell Expansion

After 42 h of culture, the cumulus expansion index (CEI) was classified according to the description provided by Jin et al. [15]: Grade 0, cumulus cells fall off and leave partially or entirely naked oocytes without cell expansion; Grade 1, cumulus cells are closely packed around oocytes without cell expansion; Grade 2, the outermost cumulus cell layers exhibit expansion; Grade 3, all layers except the corona radiata expanded obviously, cumulus cells exhibit significant expansion in all layers except the corona radiata; and Grade 4, all layers expanded.

### 2.4. Assessment of Nuclear Maturation

After 42–44 h of culture, COCs were transferred with hyaluronidase (0.1%) in a pipette, and then oocytes and cumulus cells were separated. Oocytes were classified under the bright field of a microscope into metaphase II (MII), immature, or degenerated. The sign that an oocyte reaches the MII stage is that it has the first polar body, the mature oocyte.

### 2.5. Detection of Intracellular ROS and GSH Levels

After 42–44 h, ROS and GSH levels of MII oocytes were detected through H2DCFDA (Beyotime, Shanghai, China) and CMF2HC (Beyotime, Shanghai, China). Each group was stained by H2DCFDA and CMF2HC for 10 μM and 30 min at room temperature, then 4 μL PBS droplets were placed in a 35 mm small dish (Thermo Fisher Scientific, Wilmington, DE, USA), and each group of oocytes was transferred to different dishes in turn. Stained samples were imaged with an inverted fluorescence microscope (Ti2eU; Nikon, Tokyo, Japan) using ImageJ software (version 8.0.2, NIH, Bethesda, MD, USA) to examine fluorescence intensity. For each experimental group, 30 to 60 oocytes were selected for analysis.

### 2.6. Lipid Droplet (LD), Fatty Acid (FA), and ATP Staining

After IVM, oocytes with the first polar body were selected for staining. Oocytes were fixed in 4% paraformaldehyde at 4 °C for 7 h, then transferred to DPBS containing 500 nM BODIPY-ATP (BODIPY FL ATP; A12410; Molecular Probes, Eugene, OR, USA), 10 μg/mL BODIPY-LD (BODIPY 493/503; D3922; Molecular Probes) or 6 μM BODIPY-FA (BODIPY 558/568 C12; D3835; Molecular Probes) for ATP, LDs, and FAs for 1 h. Next, oocytes were washed thrice with DPBS and transferred to a confocal dish. Lastly, oocytes were photographed using a laser confocal microscope (Leica TCS SP8; Wetzlar, Germany) and ImageJ software (version 8.0.2, NIH, Bethesda, MD, USA) to examine fluorescence intensity. Each group selected at least 40 individual oocytes for result analysis.

### 2.7. Mitochondrial Abundance and Mitochondrial Membrane Potential (MMP) Assay

MII oocytes were then incubated in DPBS containing 200 nM MitoTracker Red CMXRos (Beyotime, Shanghai, China) for 1 h to measure mitochondrial abundance. Then, oocytes were co-incubated in DPBS with 15 μg/mL JC-1 (Beyontime, Shanghai, China) for 16 h to measure MMP. An inverted fluorescence microscope (Ti2eU; Nikon, Tokyo, Japan) was used to detect fluorescence signals, and the ratio of red to green fluorescence was examined by ImageJ software (version 8.0.2, NIH, Bethesda, MD, USA).

### 2.8. Parthenogenetic Activation (PA) and In Vitro Culture (IVC)

Following the same activation procedure as Wang et al. [1], MII oocytes were placed into the activation solution. Next, parthenogenetic activation was conducted using two 160 V direct current pulses for 40 μs. After activation, embryos were transferred to a porcine zygote medium (PZM-3) containing 7.5 mg/mL cytochalasin B at 38.5 °C in a 5% CO_2_ atmosphere for 3 h. Finally, oocytes were placed in a four-well plate with 50 μL PZM-3 at 38.5 °C and 5% CO_2_ for seven days. The cleavage rate was determined on Day 2. Blastocysts on Day 7 were detected and collected, and the total cell number was detected through 5 μg/mL Hoechst-33342 for 7 min. The blastocysts were observed with an inverted fluorescence microscope (Ti2eU; Nikon, Tokyo, Japan) to count cell nuclei.

### 2.9. Reverse-Transcription-Quantitative Polymerase Chain Reaction (RT-qPCR) Analysis

The total mRNA of cumulus cells or oocytes was extracted from each group using the Dynabeads™ mRNA DIRECT™ Purification Kit (Invitrogen, Waltham, MA, USA). The first-strand cDNA was obtained through a kit (TIANGEN, Beijing, China). Next, cDNA content was detected using a Spectrophotometer (NanoDrop 2000, Thermo Fisher Scientific, Wilmington, DE, USA) to ensure the obtained cDNA was sufficient for subsequent qRT-PCR experiments. Transcript expression analysis was performed using the KAPA SYBR FAST Universal qPCR Kit (Kapa Biosystems, Cape Town, South Africa). A PCR system contains 10 μL KAPA SYBR FAST qPCR Master Mix (2X) Universal, forward primer and reverse primer 0.4 μL each, 1 μL of cDNA, and 7 μL of deionized water for a total volume of 20 μL. The amplification conditions were as follows: initial denaturation for 180 s at 95 °C, 40 cycles of denaturation for 3 s at 95 °C, annealing for 30 s at 60 °C, elongation for 20 s at 72 °C, and extension for 300 s at 72 °C. Each target transcript expression was calculated relative to the housekeeping gene *RN18s* for oocytes or *GAPDH* for cumulus cells using the R = 2^−ΔΔCt^ equation. Table S1 lists the primer sequences.

### 2.10. Statistical Analyses

The statistical analysis, which included checking for variable normality, was conducted using SPSS software (version 22.0, IBM Corp, Chicago, IL, USA), and the data were represented as the mean ± SEM. Fluorescence staining and PCR results were compared using the Student’s *t*-test. At least 30 oocytes were used for each repeated analysis. Each experiment was repeated at least thrice (R represents the number of repetitions of figure note). Differences with *p* < 0.05 were considered statistically significant.

## 3. Results

### 3.1. Chrysoeriol Upgraded Cumulus Expansion of Oocytes

**Figure 1:**
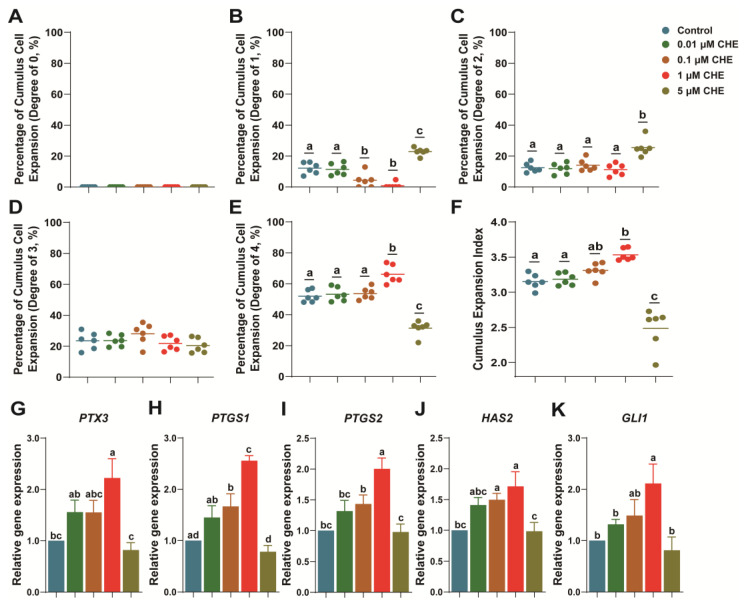
Effect of CHE on cumulus cell expansion and related transcript expression. (**A**–**F**) Different degrees and average degree of the cumulus expansion (R = 6); (**G**–**K**) expression level of cumulus expansion-related genes (R = 3). Significant differences are represented by different letters (*p* < 0.05). Shown as the average ± SEM. CHE, chrysoeriol.

We observed high-concentration inhibition during the IVM stage. Namely, when treated with 10 μM CHE in an IVM medium, the oocyte cumulus fell off, and the maturity rate was almost zero. Therefore, 5 μM was chosen as the high-concentration option to ensure a specific proportion of oocytes survived in the subsequent experiments. Our results indicated that the 1 μM CHE treatment group could significantly upregulate COCs expansion compared to the control (CEI Grade 4; 66.15 ± 2.40% vs. 51.90 ± 1.57%, respectively; Figure 1E; *p* < 0.05).

Inversely, 0.1 μM and 1 μM CHE groups significantly reduced the proportion of CEI Grade 1 in oocytes compared to the control (4.28 ± 1.93% and 0.78 ± 0.78% vs. 12.12 ± 1.52%, respectively; Figure 1B; *p* < 0.05). Notably, the 5 μM CHE group decreased the expansion ability of COCs and significantly increased the proportion of CEI Grades 1 and 2 compared to the control (CEI Grade 1: 22.83 ± 0.98% vs. 12.12 ± 1.52%; CEI Grade 2: 25.45 ± 2.29% vs. 12.43 ± 1.23%, respectively; Figure 1B,C; *p* < 0.05). In addition, the proportion of CEI Grade 4 decreased significantly (31.30 ± 1.96% vs. 51.90 ± 1.57%, respectively; Figure 1E; *p* < 0.05).

Overall, the average CEI increased significantly in the 1 μM group (3.53 ± 0.03 vs. 3.15 ± 0.04, Figure 1F; respectively; *p* < 0.05) and decreased significantly in the 5 μM group (2.48 ± 0.12 vs. 3.15 ± 0.04, respectively; Figure 1F; *p* < 0.05) compared to the control. Moreover, cumulus expansion-related transcript expressions (*PTX3*, *PTGS1*, *PTGS2*, and *HAS2*) were significantly higher in the 1 μM CHE group compared to the control (Figure 1G–J; *p* < 0.05). Furthermore, *GLI1* expression significantly increased in the 1 μM CHE group (Figure 1K; *p* < 0.05).

### 3.2. Effects of Chrysoeriol on Oocytes Development

**Figure 2:**
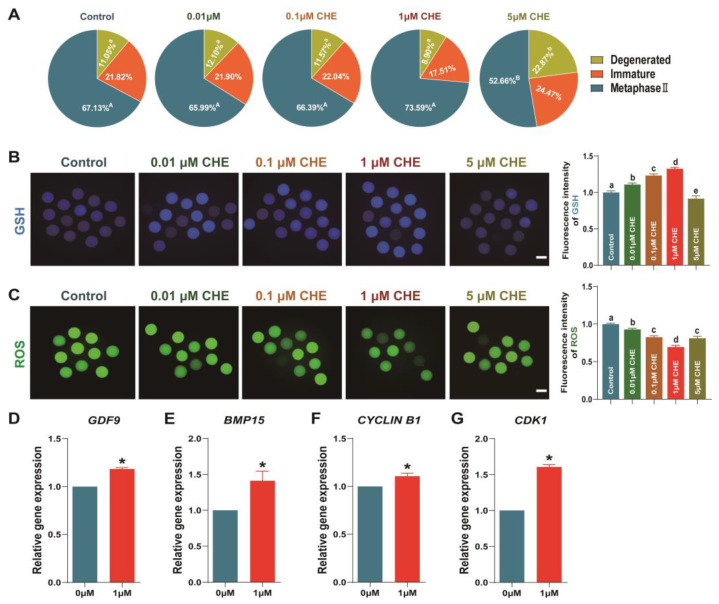
Effect of chrysoeriol on maturation and antioxidation in porcine. (**A**) Oocytes maturation, immature or degenerated rate (R = 6); (**B**,**C**) ROS and intracellular GSH levels (R = 3); (**D**–**G**) oocyte development-related transcript expressions (R = 3). Shown as the average ± SEM and significant differences are different letters or asterisks (*p* < 0.05). CHE, chrysoeriol.

As shown in Figure 2A, the 1 μM CHE treatment group exhibited an increased oocyte maturation rate, but there was no significant difference. Moreover, the degenerated oocyte proportion was significantly elevated in the 5 μM CHE group compared to the other groups (control: 11.10 ± 0.95%, 0.01 μM: 12.21 ± 0.87%, 0.1 μM: 11.70 ± 1.26%, 1 μM: 9.23 ± 1.44%, 5 μM: 22.98 ± 2.44%, respectively; *p* < 0.05), while the oocyte maturation rate was significantly decreased (52.58 ± 3.79% vs. 67.01 ± 2.18%, respectively; Figure 2A; *p* < 0.05). Excluding the 5 μM CHE group, other CHE treatment concentrations significantly increased intracellular GSH levels and lowered ROS levels in oocytes (Figure 2B,C; *p* < 0.05). The ROS and intracellular GSH levels in the 5 μM CHE group were significantly decreased compared to the control (Figure 2B,C; *p* < 0.05). Lastly, we compared the relative transcript content of oocyte development-related gene expressions between the 1 μM CHE group and the control group. *GDF9*, *CDK1*, *CYCLIN B1*, and *BMP15* were significantly higher in the 1 μM CHE group compared to the control group (Figure 2D–G; *p* < 0.05).

### 3.3. Chrysoeriol Improved Embryo Development after Parthenogenetic Activation

**Figure 3:**
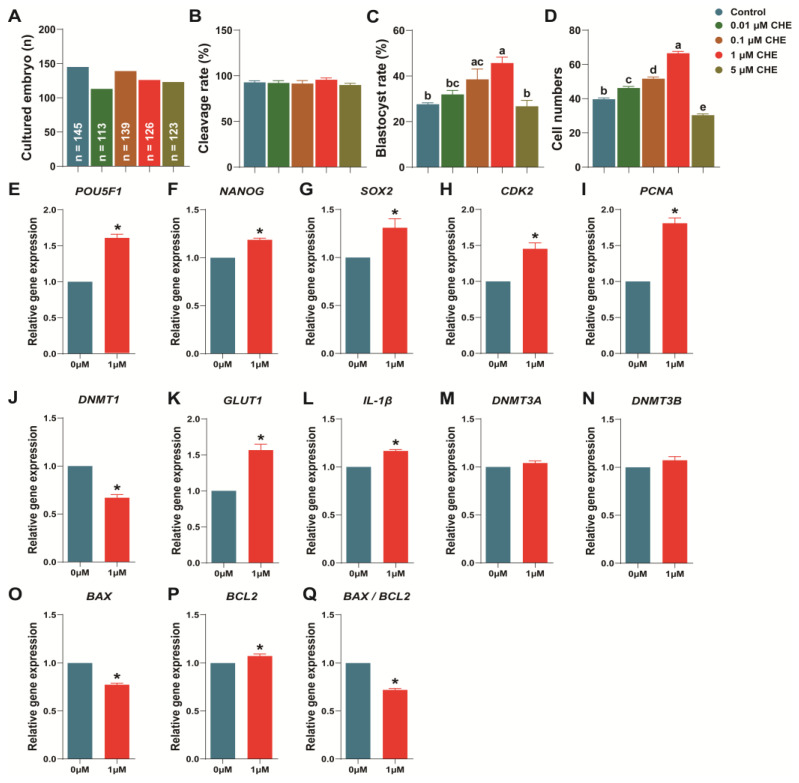
Effect of chrysoeriol on parthenogenetic-activated early embryonic development. (**A**) The total number of embryos; (**B**–**D**) embryonic development quality index (cleavage rate, blastocyst formation rate and total cell number) (R = 3); (**E**–**Q**) embryo development- and apoptosis-related transcript expressions (R = 3). Shown as the average ± SEM and significant differences are represented by different letters or asterisks (*p* < 0.05). CHE, chrysoeriol.

Results demonstrated that the parthenogenetic blastocyst formation rate treated with 0.1 μM and 1 μM CHE were significantly higher than the control group after IVC (38.52 ± 4.56% and 45.66 ± 2.59% vs. 27.61 ± 0.66%, respectively; Figure 3C; *p* < 0.05). Moreover, total cell numbers in the 5 μM CHE group decreased significantly (30.37 ± 0.74 vs. 39.67 ± 0.78, respectively; Figure 3D; *p* < 0.05), while those in other concentration groups increased (46.23 ± 0.93, 51.67 ± 1.02 and 66.57 ± 1.05 vs. 39.67 ± 0.78, respectively; Figure 3D; *p* < 0.05). However, cleavage rates did not show significant difference (Figure 3B). Based on these results, we compared the 1 μM CHE and control groups in subsequent experiments. Concerning transcript expression, our results demonstrated that the relative transcript content of embryo development-related genes (*POU5F1, CDK2, SOX2, PCNA, NANOG* and *GLUT1*) were significantly increased and *DNMT1* was significantly decreased in the 1 μM CHE group (Figure 3E–K; *p* < 0.05). Some genes related to embryo development (*DNMT3A* and *DNMT3B*) increased, but there was no significant difference (Figure 3M,N). Furthermore, the 1 μM CHE group exhibited significantly diminished *BAX* and *BAX/BCL2* ratio expressions, but *BCL2* expression significantly increased (Figure 3O–Q).

### 3.4. Chrysoeriol Participated in Lipid Homeostasis Maintenance

**Figure 4:**
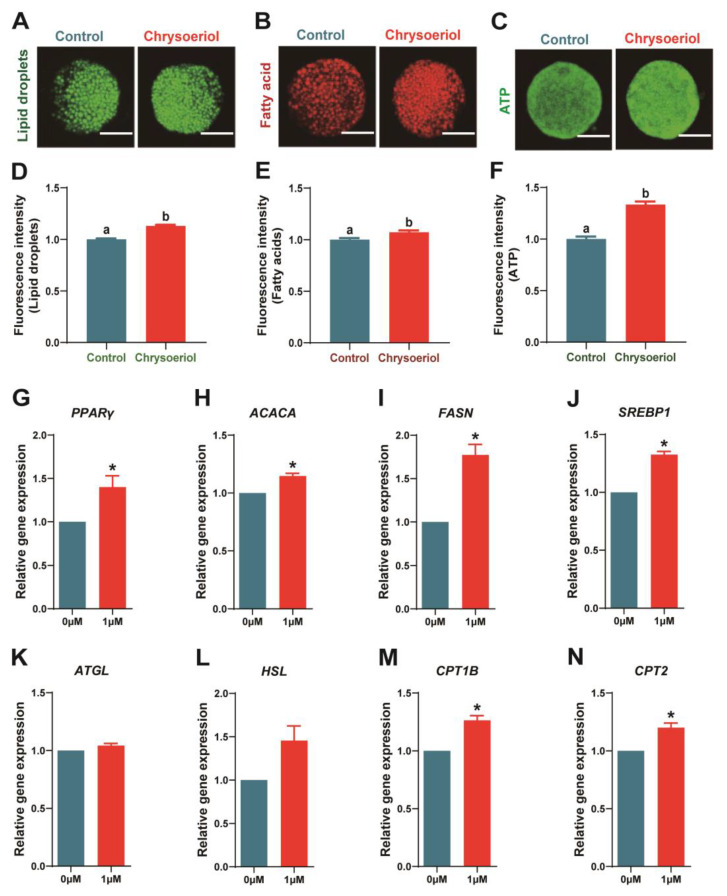
Effects of chrysoeriol on lipid metabolism. (**A**,**D**) Lipid droplet content in oocytes; (**B**,**E**) fatty acid content in oocytes; (**C**,**F**) ATP content in oocytes; (**G**–**N**) lipid metabolism-related transcript expressions. Shown as the average ± SEM of three repeated independent experiments and significant differences are represented by different letters or asterisks (*p* < 0.05). CHE, chrysoeriol.

Our results revealed that 1 μM CHE treatment significantly increased the fluorescence intensity of lipid droplets (LDs), fatty acids (FAs), and ATP compared to the control (Figure 4A–F; *p* < 0.05). In addition, lipogenesis-related transcript expressions (*PPARγ*, *ACACA*, *FASN*, and *SREBP1*) were significantly increased. Furthermore, 1 μM CHE supplementation raised lipolysis-related transcript expression levels, including *ATGL* and *HSL* (Figure 4G–L; *p* < 0.05). Moreover, β-oxidation-related (*CPT2* and *CPT1B*) transcript expressions were significantly increased in the 1 μM CHE group (Figure 4M,N; *p* < 0.05).

### 3.5. Chrysoeriol Improved the Mitochondrial Function of Oocytes

**Figure 5:**
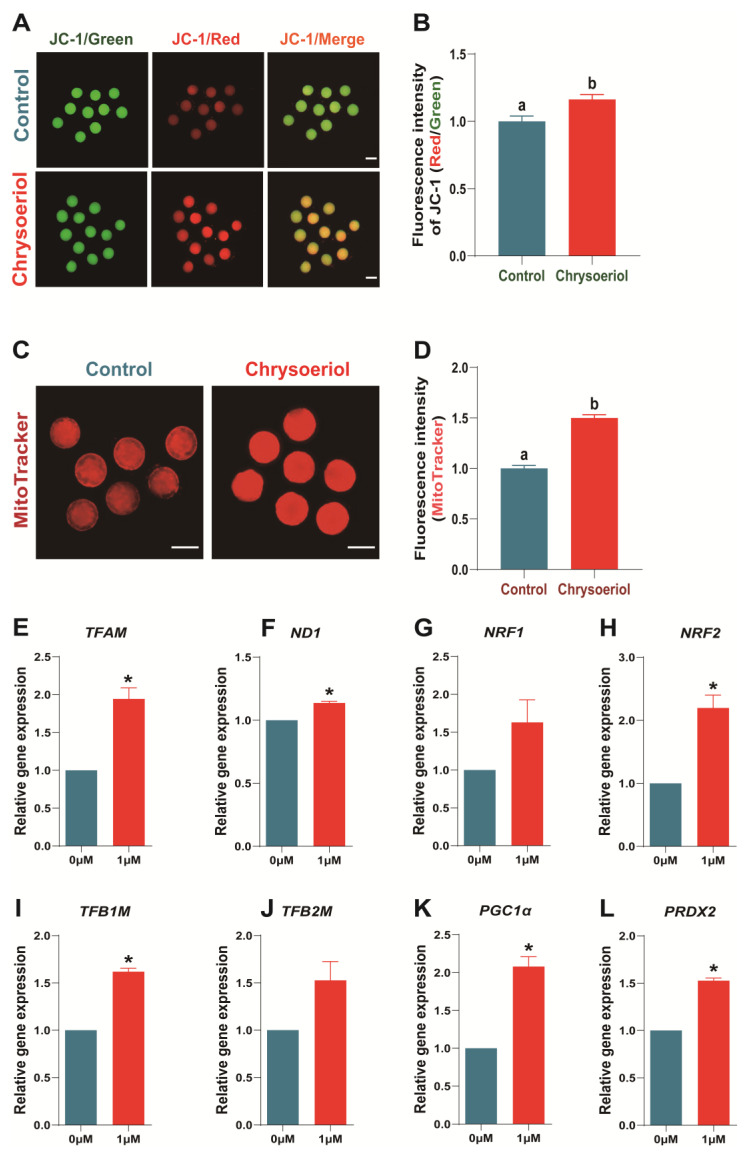
Effects of chrysoeriol on mitochondrial function in porcine oocytes. (**A**,**B**) Mitochondrial membrane potential (MMP) levels; (**C**,**D**) mitochondrial abundance; (**E**–**L**) mitochondrial function-related transcript expressions. Shown as the average ± SEM of three repeated independent experiments and significant differences are represented by different letters or asterisks (*p* < 0.05). CHE, chrysoeriol.

Abnormal lipid metabolism in oocytes often affects mitochondrial function. Therefore, we examined the effects of CHE on mitochondrial membrane potential (MMP) levels, mitochondrial abundance, and related transcript expression levels in oocytes. The 1 μM CHE treatment group displayed significantly upregulated mitochondrial abundance and MMP (Figure 5A–D; *p* < 0.05). Furthermore, 1 μM CHE supplementation significantly augmented mitochondrial function-related transcript expressions (TFAM, ND1, NRF2, TFB1M, PGC1α, and PRDX2).

## 4. Discussion

Chrysoeriol (CHE) is an antioxidant that can reduce oxidative stress. Recently, pharmacokinetic analysis in vivo has proven the CHE molecule’s stability, establishing its promising potential in preventing or treating cancer, inflammation [16], osteoporosis, diabetes, Parkinson’s disease [17], and cardiovascular diseases [18]. Previously, we determined that after the parthenogenetic activation of porcine oocytes, adding 1 μM CHE to the IVC medium improves the embryo development rate, antioxidant capacity, and mitochondrial function while reducing apoptosis and autophagy levels [1]. However, the effect of CHE on the early maturation stage of porcine oocytes remains unclear.

A recent study reported that CHE inhibited fat deposition in 3T3-L1 adipocytes and exhibited anti-adipogenic and lipolytic properties [2]. Another study confirmed that CHE can enhance the expression of antioxidant-related molecules and reduce mitochondrial damage [19]. Therefore, we demonstrated that CHE can also positively influence porcine oocyte maturation and regulate lipid metabolism and mitochondrial function. Adding CHE to an IVM medium with the same treatment concentration as the IVC stage (1 μM) significantly improved cumulus expansion, oocyte maturation rate, and blastocyst formation rate after parthenogenetic activation. In addition, cumulus expansion-, oocyte development-, or embryo development-related transcript expressions were significantly increased. Moreover, 1 μM CHE treatment decreased oxidative stress, improved mitochondrial abundance and membrane potential, and maintained lipid homeostasis by balancing lipogenesis and lipolysis. Lipid droplets (LDs), fatty acids (FAs), and ATP contents in the 1 μM CHE group increased significantly, demonstrating more substantial development potential than in the control.

Several layers of cumulus cells surround oocytes during the maturation process in vitro and are called cumulus–oocyte complexes (COCs) [20]. Cumulus cell expansion affects various fundamental changes during oocyte maturation. In porcine cells, COC volume changes are related to the outcome of oocyte maturation, fertilization, and subsequent embryo development [21,22]. Intracellular communication between cumulus cells and the oocyte is essential for numerous processes during oocyte maturation, and cumulus cells can protect oocytes from oxidative stress and reduce apoptosis [23,24,25]. Complete cumulus expansion is crucial for oocyte cytoplasm and nuclear maturation. In contrast, less or absence of cumulus cells will negatively impact the nuclear maturation rate, fertilization, and subsequent development state [21,26,27].

HAS2 is necessary for hyaluronic synthesis, and PTX3 is produced by cumulus cells during its expansion. PTX3 localizes in the matrix and exists in human cumulus cells, and HAS2 and PTX3 are necessary extracellular matrix components for female animal reproduction, which correlates with cumulus expansion [28,29,30,31]. In addition, *GLI1*, *PTGS1*, and *PTGS2* are integral for cumulus cells to transport developmental abilities to oocytes [32,33,34]. In particular, the decrease of *GLI1* may not affect oocyte maturation but will diminish developmental ability [35].

In our results, supplementation of 1 μM CHE significantly promoted the cumulus expansion and the expression of genes related to cumulus cell expansion, including *PTX3*, *PTGS1*, *PTGS2*, *HAS2*, and *GLI1*. These results were the same as supplementing exogenous melatonin [34]. However, the 5 μM CHE group exhibited opposite results, indicating that high CHE concentrations during the mature stage inhibit porcine oocyte development and beget adverse effects. We theorized that CHE is critical for porcine oocyte maturation [36].

Next, we evaluated the role of CHE on embryo development in porcine oocytes. In this experiment, we chose parthenogenetic activation to make mature oocytes continue to develop into blastocysts because the polyspermy phenomenon easily occurs in the in vitro fertilization of porcine oocytes. We found that although there was an improvement, CHE supplementation did not significantly enhance the parthenogenetic oocyte maturation, and the degradation rate was significantly increased in the 5 μM group. Furthermore, excluding the 5 μM CHE group, other groups’ relative intracellular GSH levels increased significantly. Comparatively, intracellular ROS levels significantly decreased across all CHE treatment concentrations. These results demonstrate that CHE does not significantly improve porcine oocyte maturation rates. However, as a potent antioxidant, CHE can enhance the antioxidant stress resistance of oocytes by reducing reactive oxygen species production, further supporting the theory that antioxidants can improve the microenvironment of oocyte development in vitro [37,38,39,40].

Consequently, *GDF9* and *BMP15* are secreted by oocytes, are crucial for ovarian function, and participate in regulating granulosa cells and the development quality of embryos [41,42,43]. Moreover, *CDK* and *CYCLIN B1* are related to maturation-promoting factors (MPF). Together with MPFs, these genes regulate the cell cycle through complex mechanisms, affecting mitotic and meiotic cell cycle and oocyte maturation [44,45]. Our results revealed that 1 μM CHE supplementation significantly increased *GDF9*, *BMP15*, *CYCLIN B1*, and *CDK1* transcript expressions, demonstrating that CHE can regulate and improve porcine oocyte maturation. Additionally, the 1 μM CHE treatment increased blastocyst formation rate and cell numbers but not cleavage rate. Correspondingly, 1 μM CHE treatment upregulated embryo development-related transcript expressions (Figure 3) and downregulated apoptosis-related transcript expressions. Our results indicate that CHE can help porcine oocytes resist oxidative stress and establish a stronger potential for early embryo development.

Lipids are a vital energy pool for embryo development and participate in plasma membrane synthesis. Excessive lipid droplets (LDs) formation will reduce cryotolerance and affect mitochondrial function, even the energy supply process [46]. Poor lipid metabolism during IVM will cause fatty acid (FA) accumulation and lipid droplet enlargement in oocytes and cumulus cells [9]. Our results show that LD, FA, and ATP levels in porcine oocytes treated with 1 μM CHE significantly increased compared with the control. This observation proves that CHE can increase the energy storage of oocytes. In the present study, 1 μM CHE upregulated lipogenesis-related (*PPARγ*, *ACACA*, *FASN*, and *SREBP1*), lipolysis-related (*ATGL* and *HSL*), and β-oxidation-related genes (*CPT1B* and *CPT2*). These results indicate that CHE supplementation increases energy production and balances lipid metabolism by regulating lipogenesis and lipolysis in oocytes so cells can gain stronger developmental ability.

Additionally, β-oxidation and energy production are indispensable for mitochondria, as they control intracellular Ca^2+^ homeostasis [47]. Suboptimal IVM conditions may affect mitochondrial morphology and related transcript expressions. Thus, it is essential to maintain normal mitochondrial function for oocyte maturation and development. The continuous oocyte transcription and translation require a large amount of ATP, so it is crucial to have enough functional mitochondria. On the other hand, one difference between different mitochondrial subpopulations is the membrane potential [14].

Therefore, this study confirmed the effects of 1 μM CHE on mitochondrial abundance and membrane potential in oocytes developed to the MII stage. The results indicated that 1 μM CHE supplementation significantly increased the mitochondrial content in oocytes and upregulated mitochondrial membrane potential (MMP), establishing CHE’s positive effect in improving mitochondrial function. As expected, 1 μM CHE treatment upregulated *ND1* and mitochondrial-related transcript expressions (*TFAM*, *NRF1*, *NRF2*, *TFB1M*, *TFB2M*, *PGC1α*, and *PRDX2*). In addition, *PGC1α* and *TFAM* work together to regulate mitochondrial biogenesis to stimulate mitochondrial activity [40,48]. These results demonstrated that CHE could maintain the lipid metabolism balance and improve mitochondrial function during porcine oocyte development. In addition, CHE treatment improves the early developmental potential and late embryo quality by increasing the intracellular energy content.

## 5. Conclusions

In conclusion, supplementing 1 μM CHE during IVM improved parthenogenetic oocyte maturation potential and subsequent embryo development competence by maintaining lipid homeostasis and improving mitochondrial function.

## Data Availability

All data are provided within the article and the Supplementary Materials.

## Supplementary Materials

The following supporting information can be downloaded at: https://www.mdpi.com/article/10.3390/antiox13010122/s1, Table S1: Primer sequences used for RT-qPCR.

## References

[1] Wang C.R., Ji H.W., He S.Y., Liu R.P., Wang X.Q., Wang J., Huang C.M., Xu Y.N., Li Y.H., Kim N.H. (2023). Chrysoeriol Improves In Vitro Porcine Embryo Development by Reducing Oxidative Stress and Autophagy. Vet. Sci..

[2] Song J., Lee H., Heo H., Lee J., Kim Y. (2022). Effects of Chrysoeriol on Adipogenesis and Lipolysis in 3T3-L1 Adipocytes. Foods.

[3] Ramirez G., Zamilpa A., Zavala M., Perez J., Morales D., Tortoriello J. (2016). Chrysoeriol and other polyphenols from Tecoma stans with lipase inhibitory activity. J. Ethnopharmacol..

[4] Jin J.X., Lee S., Taweechaipaisankul A., Kim G.A., Lee B.C. (2017). Melatonin regulates lipid metabolism in porcine oocytes. J. Pineal Res..

[5] Prates E.G., Nunes J.T., Pereira R.M. (2014). A role of lipid metabolism during cumulus-oocyte complex maturation: Impact of lipid modulators to improve embryo production. Mediat. Inflamm..

[6] Sturmey R.G., O’Toole P.J., Leese H.J. (2006). Fluorescence resonance energy transfer analysis of mitochondrial:lipid association in the porcine oocyte. Reproduction.

[7] Henne W.M., Reese M.L., Goodman J.M. (2018). The assembly of lipid droplets and their roles in challenged cells. EMBO J..

[8] Dunning K.R., Russell D.L., Robker R.L. (2014). Lipids and oocyte developmental competence: The role of fatty acids and β-oxidation. Reproduction.

[9] Del Collado M., da Silveira J.C., Sangalli J.R., Andrade G.M., Sousa L., Silva L.A., Meirelles F.V., Perecin F. (2017). Fatty Acid Binding Protein 3 And Transzonal Projections Are Involved in Lipid Accumulation During In Vitro Maturation of Bovine Oocytes. Sci. Rep..

[10] McEvoy T.G., Coull G.D., Broadbent P.J., Hutchinson J.S., Speake B.K. (2000). Fatty acid composition of lipids in immature cattle, pig and sheep oocytes with intact zona pellucida. J. Reprod. Fertil..

[11] Robker R.L., Akison L.K., Bennett B.D., Thrupp P.N., Chura L.R., Russell D.L., Lane M., Norman R.J. (2009). Obese women exhibit differences in ovarian metabolites, hormones, and gene expression compared with moderate-weight women. J. Clin. Endocrinol. Metab..

[12] Robker R.L., Wu L.L., Yang X. (2011). Inflammatory pathways linking obesity and ovarian dysfunction. J. Reprod. Immunol..

[13] Yang X., Wu L.L., Chura L.R., Liang X., Lane M., Norman R.J., Robker R.L. (2012). Exposure to lipid-rich follicular fluid is associated with endoplasmic reticulum stress and impaired oocyte maturation in cumulus-oocyte complexes. Fertil. Steril..

[14] Kirillova A., Smitz J.E.J., Sukhikh G.T., Mazunin I. (2021). The Role of Mitochondria in Oocyte Maturation. Cells.

[15] Jin J.X., Sun J.T., Jiang C.Q., Cui H.D., Bian Y., Lee S., Zhang L., Lee B.C., Liu Z.H. (2022). Melatonin Regulates Lipid Metabolism in Porcine Cumulus-Oocyte Complexes via the Melatonin Receptor 2. Antioxidants.

[16] Wu J.Y., Chen Y.J., Bai L., Liu Y.X., Fu X.Q., Zhu P.L., Li J.K., Chou J.Y., Yin C.L., Wang Y.P. (2020). Chrysoeriol ameliorates TPA-induced acute skin inflammation in mice and inhibits NF-κB and STAT3 pathways. Phytomedicine.

[17] Limboonreung T., Tuchinda P., Chongthammakun S. (2020). Chrysoeriol mediates mitochondrial protection via PI3K/Akt pathway in MPP+ treated SH-SY5Y cells. Neurosci. Lett..

[18] Aboulaghras S., Sahib N., Bakrim S., Benali T., Charfi S., Guaouguaou F.E., Omari N.E., Gallo M., Montesano D., Zengin G. (2022). Health Benefits and Pharmacological Aspects of Chrysoeriol. Pharmaceuticals.

[19] Kim M.H., Kwon S.Y., Woo S.Y., Seo W.D., Kim D.Y. (2021). Antioxidative Effects of Chrysoeriol via Activation of the Nrf2 Signaling Pathway and Modulation of Mitochondrial Function. Molecules.

[20] Kruip T.A., Bevers M.M., Kemp B. (2000). Environment of oocyte and embryo determines health of IVP offspring. Theriogenology.

[21] Yokoo M., Sato E. (2004). Cumulus-oocyte complex interactions during oocyte maturation. Int. Rev. Cytol..

[22] Turathum B., Gao E.M., Chian R.C. (2021). The Function of Cumulus Cells in Oocyte Growth and Maturation and in Subsequent Ovulation and Fertilization. Cells.

[23] Sutton M.L., Cetica P.D., Beconi M.T., Kind K.L., Gilchrist R.B., Thompson J.G. (2003). Influence of oocyte-secreted factors and culture duration on the metabolic activity of bovine cumulus cell complexes. Reproduction.

[24] Tatemoto H., Sakurai N., Muto N. (2000). Protection of porcine oocytes against apoptotic cell death caused by oxidative stress during In vitro maturation: Role of cumulus cells. Biol. Reprod..

[25] Walter J., Monthoux C., Fortes C., Grossmann J., Roschitzki B., Meili T., Riond B., Hofmann-Lehmann R., Naegeli H., Bleul U. (2020). The bovine cumulus proteome is influenced by maturation condition and maturational competence of the oocyte. Sci. Rep..

[26] Wongsrikeao P., Kaneshige Y., Ooki R., Taniguchi M., Agung B., Nii M., Otoi T. (2005). Effect of the removal of cumulus cells on the nuclear maturation, fertilization and development of porcine oocytes. Reprod. Domest. Anim..

[27] Zhou C.J., Wu S.N., Shen J.P., Wang D.H., Kong W.W., Lu A., Li Y.J., Zhou H.X., Zhao Y.F., Liang C.G. (2016). The beneficial effects of cumulus cells and oocyte-cumulus cell gap junctions depends on oocyte maturation and fertilization methods in mice. PeerJ.

[28] Salustri A., Garlanda C., Hirsch E., De Acetis M., Maccagno A., Bottazzi B., Doni A., Bastone A., Mantovani G., Beck Peccoz P. (2004). PTX3 plays a key role in the organization of the cumulus oophorus extracellular matrix and in in vivo fertilization. Development.

[29] Sayutti N., Abu M.A., Ahmad M.F. (2022). PCOS and Role of Cumulus Gene Expression in Assessing Oocytes Quality. Front. Endocrinol..

[30] Sugiura K., Su Y.Q., Eppig J.J. (2009). Targeted suppression of Has2 mRNA in mouse cumulus cell-oocyte complexes by adenovirus-mediated short-hairpin RNA expression. Mol. Reprod. Dev..

[31] Yung Y., Ophir L., Yerushalmi G.M., Baum M., Hourvitz A., Maman E. (2019). HAS2-AS1 is a novel LH/hCG target gene regulating HAS2 expression and enhancing cumulus cells migration. J. Ovarian Res..

[32] Adriaenssens T., Segers I., Wathlet S., Smitz J. (2011). The cumulus cell gene expression profile of oocytes with different nuclear maturity and potential for blastocyst formation. J. Assist. Reprod. Genet..

[33] da Luz C.M., da Broi M.G., Donabela F.C., Paro de Paz C.C., Meola J., Navarro P.A. (2017). PTGS2 down-regulation in cumulus cells of infertile women with endometriosis. Reprod. Biomed. Online.

[34] Lee S., Jin J.X., Taweechaipaisankul A., Kim G.A., Ahn C., Lee B.C. (2017). Melatonin influences the sonic hedgehog signaling pathway in porcine cumulus oocyte complexes. J. Pineal Res..

[35] Liu Y., Wei Z., Huang Y., Bai C., Zan L., Li G. (2014). Cyclopamine did not affect mouse oocyte maturation in vitro but decreased early embryonic development. Anim. Sci. J..

[36] Shi J.M., Tian X.Z., Zhou G.B., Wang L., Gao C., Zhu S.E., Zeng S.M., Tian J.H., Liu G.S. (2009). Melatonin exists in porcine follicular fluid and improves in vitro maturation and parthenogenetic development of porcine oocytes. J. Pineal Res..

[37] Jiang Y., Shi H., Liu Y., Zhao S., Zhao H. (2021). Applications of Melatonin in Female Reproduction in the Context of Oxidative Stress. Oxid. Med. Cell. Longev..

[38] Taweechaipaisankul A., Jin J.X., Lee S., Kim G.A., Lee B.C. (2016). The effects of canthaxanthin on porcine oocyte maturation and embryo development in vitro after parthenogenetic activation and somatic cell nuclear transfer. Reprod. Domest. Anim..

[39] Jin J.X., Lee S., Khoirinaya C., Oh A., Kim G.A., Lee B.C. (2016). Supplementation with spermine during in vitro maturation of porcine oocytes improves early embryonic development after parthenogenetic activation and somatic cell nuclear transfer. J. Anim. Sci..

[40] Yin Z., Sun J.T., Cui H.D., Jiang C.Q., Zhang Y.T., Lee S., Liu Z.H., Jin J.X. (2021). Tannin Supplementation Improves Oocyte Cytoplasmic Maturation and Subsequent Embryo Development in Pigs. Antioxidants.

[41] Du M., Fu X., Zhou Y., Zhu S. (2013). Effects of Trichostatin A on Cumulus Expansion during Mouse Oocyte Maturation. Asian-Australas. J. Anim. Sci..

[42] Peng J., Li Q., Wigglesworth K., Rangarajan A., Kattamuri C., Peterson R.T., Eppig J.J., Thompson T.B., Matzuk M.M. (2013). Growth differentiation factor 9:bone morphogenetic protein 15 heterodimers are potent regulators of ovarian functions. Proc. Natl. Acad. Sci. USA.

[43] Belli M., Shimasaki S. (2018). Molecular Aspects and Clinical Relevance of GDF9 and BMP15 in Ovarian Function. Vitam. Horm..

[44] Li J., Zhang H.Y., Wang F., Sun Q.Y., Qian W.P. (2021). The Cyclin B2/CDK1 Complex Conservatively Inhibits Separase Activity in Oocyte Meiosis II. Front. Cell Dev. Biol..

[45] Li J., Qian W.P., Sun Q.Y. (2019). Cyclins regulating oocyte meiotic cell cycle progression. Biol. Reprod..

[46] de Andrade Melo-Sterza F., Poehland R. (2021). Lipid Metabolism in Bovine Oocytes and Early Embryos under In Vivo, In Vitro, and Stress Conditions. Int. J. Mol. Sci..

[47] Rossi A., Pizzo P., Filadi R. (2019). Calcium, mitochondria and cell metabolism: A functional triangle in bioenergetics. Biochim. Biophys. Acta Mol. Cell Res..

[48] You Y., Hou Y., Zhai X., Li Z., Li L., Zhao Y., Zhao J. (2016). Protective effects of PGC-1α via the mitochondrial pathway in rat brains after intracerebral hemorrhage. Brain Res..

