# Supplementary material for: Chrysoeriol Improves the Early Development Potential of Porcine Oocytes by Maintaining Lipid Homeostasis and Improving Mitochondrial Function
Source: Antioxidants (Basel). 2024 Jan 19;13(1):122. doi: 10.3390/antiox13010122 (PMC10812720; doi:10.3390/antiox13010122)
Supplement: Supplementary file 1 [file antioxidants-13-00122-s001.zip › antioxidants-2781730-supplementary.pdf]

TableS1:Primer sequences used for real-time PCR

Genes	Primer sequences (5'-3')	Base
GAPDH	F:GTCGGTTGTGGATCTGACCT	20
	R:TTGACGAAGTGGTCGTTGAG	20
RN18s	F:TCCAATGGATCCTCGCGGAA	20
	R:GGCTACCACATCCAAGGAAG	20
PTX3	F:GGCCAGGGATGAATTTTAC	19
	R:GCTATCCTCTCCAACAAGTGA	21
PTGS1	F:CAACACGGCACACGACTACA	20
	R:CTGCTTCTTCCCTTTGGTCC	20
PTGS2	F:ACAGGGCCATGGGGTGGACT	20
	R:CCACGGCAAAGCGGAGGTGT	20
HAS2	F:AGTTTATGGGCAGCCAATGTAGTT	24
	R:GCACTTGGACCGAGCTGTGT	20
GLI1	F:AGAGGGACAGCTCTGAACAC	20
	R:GCTACGTCTCTTCCTCCTGA	20
GDF9	F:CAGTCAGCTGAAGTGGGACA	20
	R:TGGATGATGTTCTGCACCAT	20
BMP15	F:CCTCCATCCTTTCCAAGTCA	20
	R:GTGTAGTACCCGAGGGCAGA	20
CYCLIN B1	F:CAACTGGTTGGTGTCACTGC	20
	R:TTCCATCTGCCTGATTTGGT	20
CDK1	F:GGGCACTCCCAATAATGAAGT	21
	R:GTTCTTGATACAACGTGTGGGAA	23
POU5F1	F:TTTGGGAAGGTGTTCAGCCAAACG	24
	R:TCGGTTCTCGATACTTGTCCGCTT	24
NANOG	F:GGTTTATGGGCCTGAAGAAA	20
	R:GATCCATGGAGGAAGGAAGA	20
SOX2	F:ATGCACAACTCGGAGATCAG	20
	R:TATAATCCGGGTGCTCCTTC	20
CDK2	F:TGTGCGAGTGGATGCGGAAG	20
	R:CCGAATGGTGATGTAGCGAC	20
PCNA	F:CCTGTGCAAAAGATGGAGTG	20
	R:GGAGAGAGTGGAGTGGCTTTT	21
DNMT1	F:TCGAACCAAAACGGCAGTAC	20
	R:CGGTCAGTTTGTGTTGGACA	20
GLUT1	F:GCTTCCAGTATGTGGAGCAA	20
	R:AAGCAATCTCATCGAAGGTC	20
1L-1β	F:GCCAATGGTTTTCTCTGTGATGCC	24
	R:CTCATGCAGAACACCACTTCTCTC	24
DNMT3B	F:AGTGTGTGAGGAGTCCATTG	20
	R:GCTTCCGCCAATCACCAGT	19

BAX	F:TGCCTCAGGATGCATCTACC	20
	R:AAGTAGAAAAGCGCGACCAC	20
BCL2	F:AGGGCATTCAGTGACCTGAC	20
	R:CGATCCGACTCACCAATACC	20
PPARγ	F:AGAGCTGATCCAATGGTTGC	20
	R:GAGTTGGAAGGCTCTTCGTG	20
ACACA	F:AACAAGGACCTGGTGGAGTG	20
	R:GTCATGTGCACGATGGAATC	20
FASN	F:AACTTCCGAGACGTCATGCT	20
	R:GTGCTGAAGCAGCAGAACAG	20
SREBP1	F:ACCCGCTTCTTCCTGAGTA	19
	R:ACGGAACAACTGAGTCACCT	20
ATGL	F:CGAACTCAAGAGCACCATCA	20
	R:TTGCACATCTCTCGAAGCAC	20
HSL	F:TGTCTTTGCGGGTATTCG	18
	R:TTGTGCGGAAGAAGATGC	18
CPT1B	F:ATCAAGCCTGTGATGGCTCT	20
	R:GAGCCACACCTTGAAGAAGC	20
CPT2	F:AGTTCCAGAGAGGAGGCAAAG	21
	R:GAGCATCTCTTGGTGAAGACG	21
TFAM	F:TCCGTTCAGTTTTGCGTATG	20
	R:TTGTACACCTGCCAGTCTGC	20
ND1	F:TCCTACTGGCCGTAGCATTCCT	22
	R:TTGAGGATGTGGCTGGTCGTAG	22
NRF1	F:ACCATCCAGACAACGCAA	18
	R:ACTCCAGTAAGTGCTCCGAC	20
NRF2	F:GCCCAGTCTTCATTGCTCCT	20
	R:AGCTCCTCCCAAACTTGCTC	20
TFB1M	F:CGAGGGCTTGGAATGTTA	18
	R:CGTGTGCCTGAGTTCTTCT	19
TFB2M	F:GCAAGGAGGAAGGATGTT	18
	R:CAAGTAATGCTCGTGTCAGG	20
PGC1α	F:TTCCGTATCACCACCCAAAT	20
	R:ATCTACTGCCTGGGGACCTT	20
PRDX2	F:ATGGTGTGCTGAAGGAAGATGAAGG	25
	R:CATGCTCGTCTGTGTACTGGAAGG	24

F:Forward primer; R:Reverse primer
